# Antioxidant Properties of Casein Phosphopeptides (CPP) and Maillard-Type Conjugated Products

**DOI:** 10.3390/antiox9080648

**Published:** 2020-07-22

**Authors:** Huiying Zhang, Soichiro Nakamura, David D. Kitts

**Affiliations:** 1 Food, Nutrition and Health Program, Faculty of Land and Food Systems, the University of British Columbia, Vancouver, BC V6T 1Z4, Canada; amelie.huiying.zhang@ubc.ca; 2 Department of Agriculture, Graduate School of Science and Technology, Shinshu University, 8304 Minamiminowamura, Kamiina, Nagano 399-4598, Japan; snakamu@shinshu-u.ac.jp

**Keywords:** casein phosphopeptides, CPP, peptide–polysaccharide conjugates, transition metal quenching activity, free radical scavenging antioxidant activity

## Abstract

A casein phosphopeptide (CPP) fraction derived from tryptic hydrolysis of bovine casein was evaluated for antioxidant activity. Conjugations or mixtures of CPP with polysaccharide, galactomannan (Gal), or xyloglucan (Xyl) were prepared to evaluate potential enhancement of CPP antioxidant activity. The effect of calcium was also investigated. The CPP preparation alone was effective at scavenging hydroxyl radicals and sequestering Fe^2+^ to protect against Fenton reaction-induced deoxyribose oxidation in non-site-specific (up 63.3% inhibition) and site-specific (up 32.1% inhibition) binding assays, respectively. CPP also effectively quenched 2,2’-azinobis-3-ethylbenzothiazoline-6-sulfonic acid radicals (ABTS^•+^) to an extent of 67.6% scavenging in an aqueous system. In a soybean lecithin liposome system, CPP exhibited effective protection against peroxyl radical-induced liposomal peroxidation (38.3% of control in terms of rate of propagation). Conjugating CPP with Gal or Xyl polysaccharides using Maillard reaction conditions significantly reduced activity in the Fenton reaction-deoxyribose assays, while exhibiting no effect on the antioxidant activity of native CPP in both the ABTS and liposome assays, respectively. These results represent comparative antioxidant capacity of the native CPP and associated conjugates in phases that varied in relative hydrophilic and hydrophobic character. We conclude that CPP has the potential to act as both a primary and secondary antioxidant by displaying transition metal ion sequestering activity and free radical quenching activity. Improvements in antioxidant activity of CPP by Maillard-type conjugation with Xyl or Gal were relatively small and model-specific.

## 1. Introduction

The success of functional foods is based on the stability of targeting bioactive components in formulations and the use of technologies that will optimize the expected desired beneficial properties. Incorporation of antioxidant additives into product formulations or food package materials are examples of strategies that extend product quality and organoleptic shelf-life, which otherwise is lost due to oxidation reactions. Indeed, the functional properties of casein have been studied to reduce food spoilage and plastic waste [1].

Major protein fractions present in human and bovine milk (casein and whey) and some milk protein-derived peptides have been reported to have antioxidant activity [2,3,4,5,6,7]. In particular, specific casein hydrolysates from bovine milk demonstrate antioxidant activity via radical scavenging properties in both aqueous and lipid model systems [8,9,10,11,12,13]. Casein phosphopeptides (CPPs) derived from tryptic digests of casein also have a strong affinity to sequester divalent metal ions, such as non-heme iron and copper, two potential food pro-oxidants under specific conditions [14,15,16]. Bovine milk casein-derived CPPs may therefore serve to remove pro-oxidative metal catalysts such as iron from an environment of oxidizable lipids to prevent lipid oxidation [17]. Phosphoserine clusters on CPPs bind divalent metal ions through ionic bonding and are believed to be important constituents for chelating and solubilizing the ions [18,19,20]. The metal-chelating activity of CPPs has been shown to be useful in preventing autoxidation of lipids and improving oxidative stability of processed beef [19]. A specific CPP, β-caseinophosphopeptide (f1-25), was found to prevent oxidation of muscle phospholipids induced by iron/ascorbate combination [21]. Although the role of phosphoseryl residues in eliciting such response is somewhat elucidated, there is less information on whether the antioxidant activity of CPPs could be improved by enriching for this characteristic.

**Figure 1:**
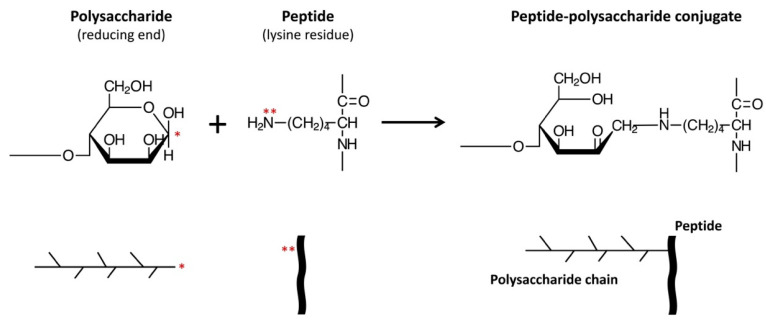
General principle for constructing peptide (e.g., CPP)–polysaccharide (e.g., galactomannan) conjugates using Maillard Reaction [25]. * reducing-end carbonyl group of galactomannan; ** free amino group (e.g., lysine) or N-terminal amino acid.

Moreover, various functional properties of proteins have been shown to be improved by conjugation with polysaccharides or oligosaccharides [22,23]. The conjugation reaction involves covalently binding the ε-amino groups of the protein to the reducing-end carbonyl groups of the sugar, under controlled heating and humidity conditions, similar to that in a Maillard reaction (Figure 1). These protein–saccharide conjugates are also known as Maillard-type neoglycoproteins or neoglycoconjugates [24]. In Figure 1, we present the concept of applying the Maillard reaction using a polysaccharide to conjugate a peptide (CPP).

The emulsifying and antioxidant properties of proteins have been reported to be greatly improved by such conjugations. Milk protein–carbohydrate conjugation achieved using the Maillard reaction produces products with techno-functional properties [26]. For example, conjugation of 2’-fucosyllactose, a human milk oligosaccharide, to α-lactalbumin significantly increases reducing power and antioxidant activity [27]. Dextran conjugation was shown to enhance emulsification power of soy protein β-conglycinin [28] and whey protein isolate [29,30], as well as improving free radical scavenging power and antioxidant activities of soy protein isolate [31]. In addition to dextran, antioxidant activity of ovalbumin [32,33], antioxidant and antimicrobial activity of lysozyme [33,34], and emulsifying and antioxidant properties of phosvitin [25,35] were all significantly enhanced through conjugation reactions of the proteins with galactomannans, a group of polysaccharides derived from the cell walls of leguminous plants and *Aspergillus* spp. fungi [36].

Bovine milk β-casein has a higher surface activity and superior adhesion to oil-in-water emulsions than milk whey protein β-lactoglobulin, egg yolk phosvitin, and soy protein β-conglycinin, resulting in a higher oxidative stability of the emulsion [37,38]. The oxidative stability of conjugated proteins further increases the thickness and steric stabilization of the interfacial layer, producing a physical barrier that restricts access of pro-oxidants to oxidation-sensitive components such as lipids and lipid-soluble compounds. It was of interest to determine if conjugation of saccharides to CPPs could also serve to amplify the hydrophilicity of the newly formed glycopeptide complex, thereby possibly improving the functional metal sequestering and free radical scavenging activities formerly shown in both aqueous and emulsion systems [10,11,12]. Since these peptide–saccharide conjugates are neither toxic nor mutagenic on ingestion [24,33], they have potential for safe use as value-added functional ingredients in food systems that require antioxidant protection. With proven nutritional benefits [39], in addition to potential antioxidant benefits [9], CPPs could have other roles, such as bioactive ingredients carried in edible casein coatings to preserve packaged foods. The purpose of the present study was to reproduce and expand the evidence for CCP antioxidant activity in both polar and non-polar phases, and to determine if using glycosylation products of CPP with different saccharides would improve or impair subsequent antioxidant activity relevant to these distinct phases.

## 2. Materials and Methods

### 2.1. CPP, Polysaccharides, and CPP–Polysaccharide Conjugates

CPP was prepared by dissolving bovine milk casein in water making a 10% solution, with a pH adjusted to 8.0. Crystalline trypsin from porcine pancreas was added to a 0.01% (*w/v*) final concentration against the substrate and incubated at 50 °C for 6 h. The pH of the casein hydrolysate was adjusted to pH 4.5 before precipitating the phosphopeptides by adding calcium chloride (1.1% *w/v*) and ethanol (50% *v/v*).

Galactomannan (Gal), a mannase hydrolysate of guar gum (Taiyo Chemicals Co., Ltd., Gobo, Wakayama, Japan), and xyloglucan (Xyl), a β-glucanase hydrolysate of tamarind seed (Sumitomo Dainippon Pharma Co., Ltd., Osaka, Osaka, Japan), were selected as the two polysaccharides for CPP conjugation. Polysaccharides were mixed in water at a weight ratio of 1:1 and lyophilized. The dry polysaccharides were individually added to CPP in excess amount and incubated for 3 days at 60 °C under 65% relative humidity. The incubated samples were applied on an open column (1.6 × 5.5 cm) of DEAE-Toyopearl (Tosoh Corporation, Tokyo, Japan), equilibrated with 20 mM phosphate buffer (pH 7.4), and the column was washed with the same buffer to separate free carbohydrates. The CPP conjugates were eluted with 500 mM NaCl buffer (pH 7.4). Peak fractions were collected together and applied to an open column with Sephacryl S-300HR (GE Healthcare, Chicago, IL, USA). The conjugates were eluted with 0.1 M phosphate buffer (pH 7.4). Concentration of protein was directly determined by absorbance at 280 nm, while that of carbohydrate was determined by the absorbance at 470 nm after color development using phenol-sulfuric acid reaction. The molar binding ratio was 1:1.9 between CPP and Gal and 1:2.2 for CPP and Xyl.

Calcium-free CPP conjugate samples were prepared by dissolving 100 mg CPP conjugates in 10 mM ethylenediaminetetraacetic acid (EDTA) and stirring the mixture for 2 h at room temperature. The mixture was dialyzed against deionized water for 2 days at 4 °C using Spectra/Por MWCO 1000 (Spectrum Labs Inc., Rancho Dominquez, CA, USA).

### 2.2. Molecular Weight

Casein and CPP were analyzed by electrophoresis using 16.5% acrylamide Tris-Tricine (N-tris [hydroxymethyl] methyl glycine) SDS (sodium dodecyl sulfate) ready gel according to Laemmli [40], and a Mini-Protean I Mini-Cell slab gel electrophoresis unit (Bio-Rad Laboratories, Inc., Richmond, CA, USA). A 4% acrylamide stacking gel (pH 6.8) was used. Protein samples (40 μg) and polypeptide molecular standards were heated at 100 °C for 5 min in 1.0 M Tris-tricine buffer, pH 6.8 containing 10% SDS. Electrophoresis was performed at a constant voltage of 100 V for 100 min with a Tris-tricine running buffer. Gels were placed in 40% methanol and 10% acetic acid fixative solution for 30 min, stained in 0.025% Coomassie Blue G-250 solution (with 10% acetic acid) for 1 h and destained in 10% acetic acid for 3 × 15-min destain washes.

### 2.3. Determination of Antioxidant Capacity of CPP Preparation

ORAC (oxygen radical absorption capacity) assay was employed to initially determine the antioxidant capacity of the CPP fraction used in this study, with modified method of Dávalos et al. [41]. Briefly, CPP was dissolved in 75 mM phosphate buffer (pH 7.0) and 60 nM fluorescein in 96-well plate at 37 °C and the reaction was initiated by 12 mM peroxyl radical initiator 2,2’-azobis (2-methylpropionamidine) dihydrochloride (AAPH). Fluorescence (excitation = 485 nm; emission = 527 nm) was monitored every minute for 60 min using fluorescent microplate reader (Fluoroskan Ascent FL, Thermo LabSystems Inc., Helsinki, Finland) and area under curve (AUC) was plotted against sample concentration. An AUC–concentration regression was obtained from the calibrator Trolox. The ORAC value was calculated from the ratio between slope of sample and slope of Trolox and expressed as μmol of Trolox equivalent per mg sample.

### 2.4. Hydroxyl Radical Scavenging Assay in a Deoxyribose Model

A Fenton reaction model containing 0.1 mM of Fe^3+^ as the catalytic metal was used [42]. The substrate 2-deoxyribose (3.6 mM) was mixed together with CPP test samples at concentrations of 0.05, 0.10, 0.50, and 1.0 mg CPP/mL. Other components of the assays included 0.1 mM EDTA (for the non-site-specific assay only), 0.1 mM ferric chloride, 0.1 mM ascorbic acid, and 1 mM H_2_O_2_. The reaction mixture was incubated at 37 °C for 1 h. Following incubation, 1 mL of 10% (*v/v*) trichloroacetic acid (TCA) and 1% 2-thiobarbituric acid (TBA) were added and the mixture was boiled for 15 min at 95 °C. Absorbance at 532 nm was recorded after cooling. The extent of protection from deoxyribose degradation by hydroxyl radicals generated by the Fenton reaction was calculated using the equation:

(1) $$\%\mathrm{Inhibition}=\frac{\mathrm{Abs}@532{\mathrm{nm}}_{\mathrm{control}}-\mathrm{Abs}@532{\mathrm{nm}}_{\mathrm{sample}}}{\mathrm{Abs}@532{\mathrm{nm}}_{\mathrm{control}}}\times 100$$

### 2.5. Free Radical Scavenging Assay

The ability of CPP and conjugates at different concentrations (0.05, 0.10, 0.50, and 1.00 mg CPP/mL) to scavenge stable 2,2’-azinobis-3-ethylbenzothiazoline-6-sulfonic acid (ABTS) radical cations, ABTS^•+^, was investigated as described by Liang et al. [43]. Discoloration was determined by comparing the absorbance at 734 nm of the treatment groups with that of the control after 8-min incubation at room temperature. The inhibitory percentage of ABTS was calculated according to the following equation:

(2) $$\%\mathrm{Scavenging}=\frac{\mathrm{Abs}@734{\mathrm{nm}}_{\mathrm{control}}-\mathrm{Abs}@734{\mathrm{nm}}_{\mathrm{sample}}}{\mathrm{Abs}@734{\mathrm{nm}}_{\mathrm{control}}}\times 100$$

### 2.6. Peroxyl Radical Scavenging Assays in a Liposome Model

Liposomes were made by sonicating soybean lecithin (L-α-phosphatidylcholine) in 10 mM phosphate buffer (pH 7.4) in an ice-water bath for 20 min [44]. Peroxyl radical-induced liposomal peroxidation was conducted at a constant temperature of 37 °C using an ATI Unicam (UV2) UV/Vis spectrophotometer (Thermo Fisher Scientific, Waltham, MA, USA). The reaction was initiated by the addition of 0.2 mM AAPH to a mixture of 0.1 mg/mL of liposome in 10 mM phosphate buffer (pH 7.4) which already included CPP and conjugate samples at concentrations of 0.05, 0.10, and 0.50 mg CPP/mL. Trolox (1.0 μg/mL) was tested as a positive control. Generation of conjugated diene hydroperoxide was monitored by taking absorbance readings at 234 nm every 4 min for 100 min, and the rate of propagation was calculated by the application of linear regression on kinetic graphs. The efficacy of samples against liposome peroxidation was compared to that of control and expressed as percent of control.

### 2.7. Statistical Analysis

All data (with the exception of the liposome assays) were collected in triplicates and analyzed by one-way ANOVA (α ≤ 0.05), followed by a multiple-range Tukey’s post-hoc test using the GraphPad Prism Analysis software (GraphPad Software Inc., San Diego, CA, USA) to identify significant differences among treatment means (*p* ≤ 0.01). Data were obtained from three different individual experiments for the liposome assays and submitted to linear regression using GraphPad software. Rates of propagation (i.e., slope of regression equation) were then analyzed by one-way ANOVA (α ≤ 0.05) and Tukey’s test (*p* ≤ 0.01).

## 3. Results

### 3.1. Recovery of CPP

**Figure 2:**
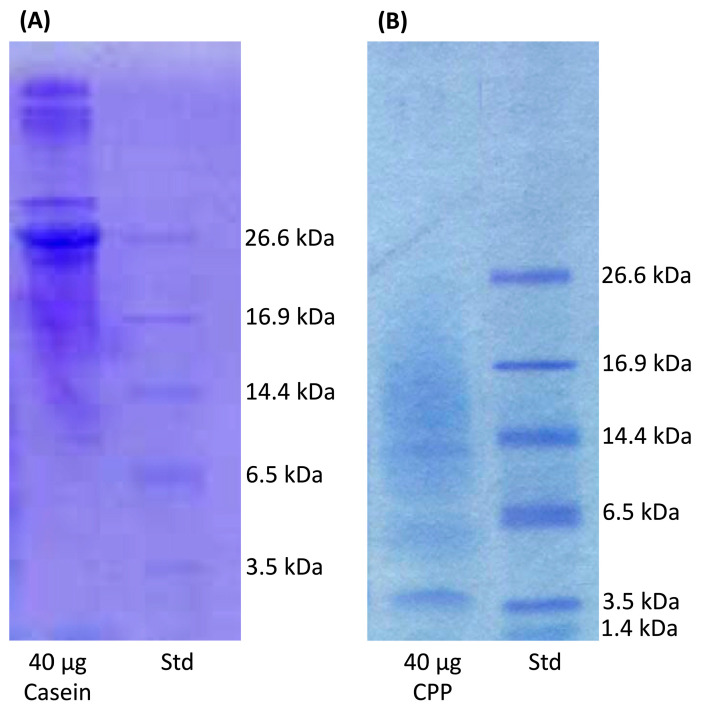
A 16.5% Tris-Tricine SDS-PAGE Gel stained with Coomassie G-250 using the Laemmli buffer system (1.0 M Tris, pH 8.45). Std, Polypeptide Standards. A comparison of (**A**) casein versus. (**B**) CPP is presented to the show CPP from the tryptic digested casein.

The use of a tricine-sodium dodecyl sulfate-polyacrylamide gel electrophoresis, TSDS-PAGE [45] enabled identification of CPP fractions recovered from tryptic digestion of casein (Figure 2). A notable casein peptide in crude hydrolysate was detected at molecular weight approximate to 3.5 kDa. This result corresponds to other reports that identified CPP contained peptides corresponding to the 2.6-kDa α_s2_-casein (2-21)-4P [46], the 2.7-kDa α_s1_-casein (59-79)-5P [46,47] or the 3.125-kDa β-casein (1-25)-4P [48].

### 3.2. ORAC Analysis

The CPP preparation used in this study exhibited an ORAC antioxidant capacity of 185 μmol Trolox equivalent/mg CPP. Free radicals vary in relative affinity to interact with atoms that have the greatest electron density. The peroxyl radical used for ORAC testing in this study has been correlated with the reducing capacity of CPP [49].

### 3.3. Site-Specific and Non-Site-Specific Scavenging Activities

**Figure 3:**
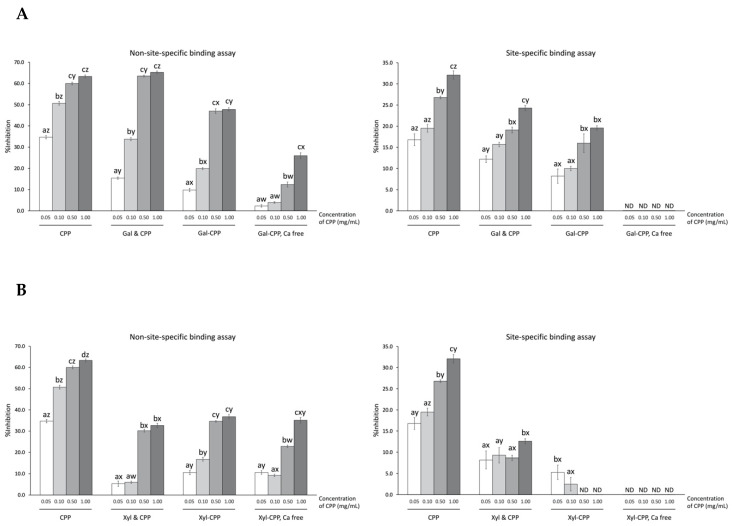
Inhibition of Fenton reaction-induced deoxyribose oxidation via non-site-specific and site-specific binding by CPP and CPP–polysaccharide ((**A**) Gal; and (**B**) Xyl) conjugates and mixtures. Non-site-specific binding assay: ascorbic acid-mediated Fenton reaction in the presence of EDTA; site-specific binding assay: ascorbic acid-mediated Fenton reaction in the absence of EDTA. CPP, casein phosphopeptide; Gal (or Xyl) & CPP, non-conjugated mixtures of galactomannan (or xyloglucan) and CPP; Gal–CPP or Xyl–CPP, conjugates of galactomannan or xyloglucan with CPP formed by employing Maillard reaction conditions; ND, not detectable (<0.1% inhibition). All detectable results are expressed as mean ± SD (*n* = 3) and were statistically analyzed by one-way ANOVA and Tukey’s post-hoc test. Letters (a–d) denote significantly different means within the same treatment group in an assay, indicating effects of CPP concentration; letters (w–z) denote significantly different means with the same CPP concentration level in an assay, indicating effects of treatment (*p* < 0.01).

CPP exhibited a concentration-dependent protection against Fenton reactant-induced deoxyribose oxidation using both site-specific and non-site-specific formats, i.e. through metal sequestering and hydroxyl radical scavenging, respectively (Figure 3). Greater (*p* < 0.01) inhibition of oxidative degradation of deoxyribose by CPP occurred by hydroxyl radical scavenging activity (e.g., non-site-specific assay yielding more than 60% inhibition at 1.00 mg/mL CPP) than by ferrous ion chelation (e.g., site-specific assay yielding up to 32% inhibition). 

Both Gal and Xyl alone had low inhibition (e.g., <2% activity in either assay; data not shown). In fact, both site-specific and non-site-specific inhibitions of deoxyribose degradation were dramatically lower for the CPP–polysaccharide conjugates, compared to the CPP alone, over the experiment concentration range (Figure 3). Gal–CPP conjugates inhibited, on average, 10–20% less deoxyribose oxidation than the corresponding non-glycosylated CPP. Similarly, Xyl–CPP conjugates were 10–30% less effective compared to non-glycosylated CPP. The simple addition of Gal or Xyl to CPP represented a non-glycosylated control mixture. The mixture of Gal and CPP produced a scavenging activity of hydroxyl radicals higher (*p* < 0.01) than Gal–CPP conjugate at all concentrations tested, as well as a higher (*p* < 0.01) iron chelating activity at all concentrations other than 0.50 mg/mL. Xyl and CPP mixture had a relatively lower (*p* < 0.01) hydroxyl radical scavenging activity but a higher (*p* < 0.01) ferrous iron chelation. Overall, conjugation or addition of Gal or Xyl to CPP did not result in superior antioxidant activity than CPP alone.

Removal of calcium from CPP with EDTA treatment resulted in markedly lower (*p* < 0.01) protection of deoxyribose for both Gal–CPP or Xyl–CPP conjugates at all concentrations tested in the site-specific assay and complete loss of protection (< 1% inhibition, not detectable) in the non-site-specific assay.

### 3.4. Scavenging Activity of ABTS Radicals by CPP and Conjugate Reactants

**Figure 4:**
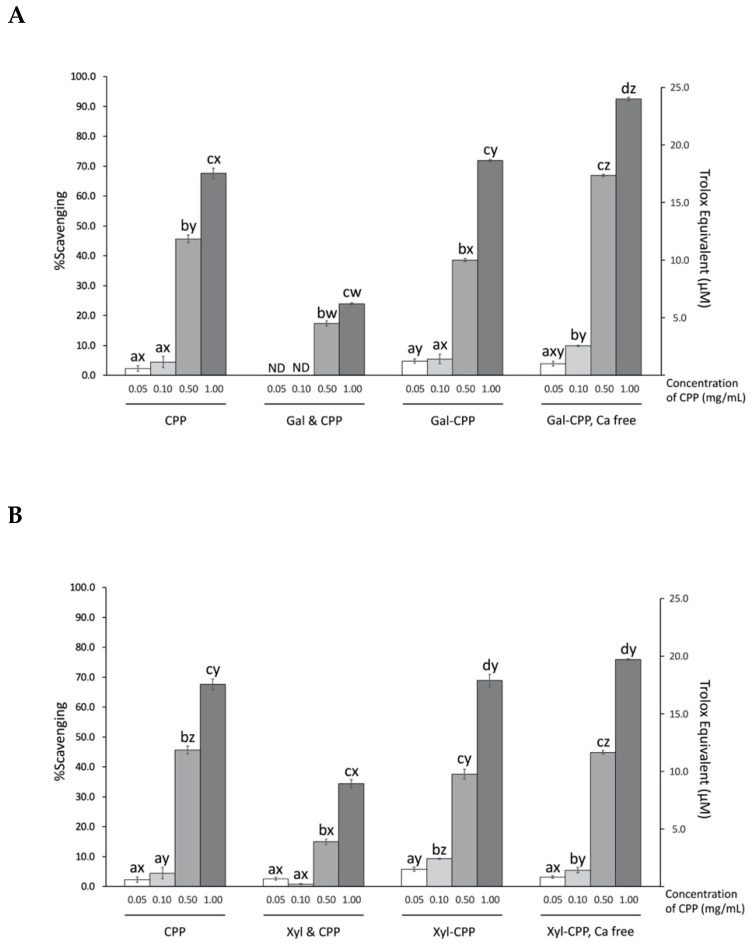
ABTS radical scavenging activity by CPP and CPP–polysaccharide ((**A**) Gal; and (**B**) Xyl) conjugates and mixtures. Trolox equivalent was calculated using the standard curve equation y = 3.896x−0.5844, where x stands for micromolar (μM) of Trolox and y for percent scavenging. CPP, casein phosphopeptide; Gal (or Xyl) & CPP, non-conjugated mixtures of galactomannan (or xyloglucan) and CPP; Gal–CPP or Xyl–CPP, conjugates of galactomannan or xyloglucan with CPP formed by employing Maillard reaction conditions; ND, not detectable (<0.1% scavenging). All detectable results are expressed as mean ± SD (*n* = 3) and were statistically analyzed by one-way ANOVA and Tukey’s post-hoc test. Letters (a–d) denote significantly different means within the same treatment group, indicating effects of CPP concentration; letters (w–z) denote significantly different means within the same CPP concentration in an assay, indicating effects of treatment (*p* < 0.01).

A concentration-dependent quenching affinity of CPP, conjugates, and non-conjugated mixtures for the aqueous, stable free radical ABTS^•+^ is shown in Figure 4. At the highest concentration tested (i.e., 1.0 mg/mL), CPP quenched 67.6% of the radicals ABTS^•+^, equivalent to 17.5 µM Trolox. Overall, conjugation of polysaccharides Gal or Xyl did not have an effect at improving the radical stabilizing activity of CPP. However, the calcium-free preparations of the Gal–CPP conjugates at higher concentrations (0.10, 0.50, and 1.00 mg/mL) showed significantly (*p* < 0.01) higher scavenging activity of ABTS radicals, while the removal of calcium from Xyl–CPP did not show such effect. The simple addition without conjugation of Gal or Xyl to the CPP preparations, on the other hand, impaired the ABTS radical scavenging ability of CPP (*p* < 0.01).

### 3.5. Effect of CPP and Conjugate Reactants on the Liposome Peroxidation

**Figure 5:**
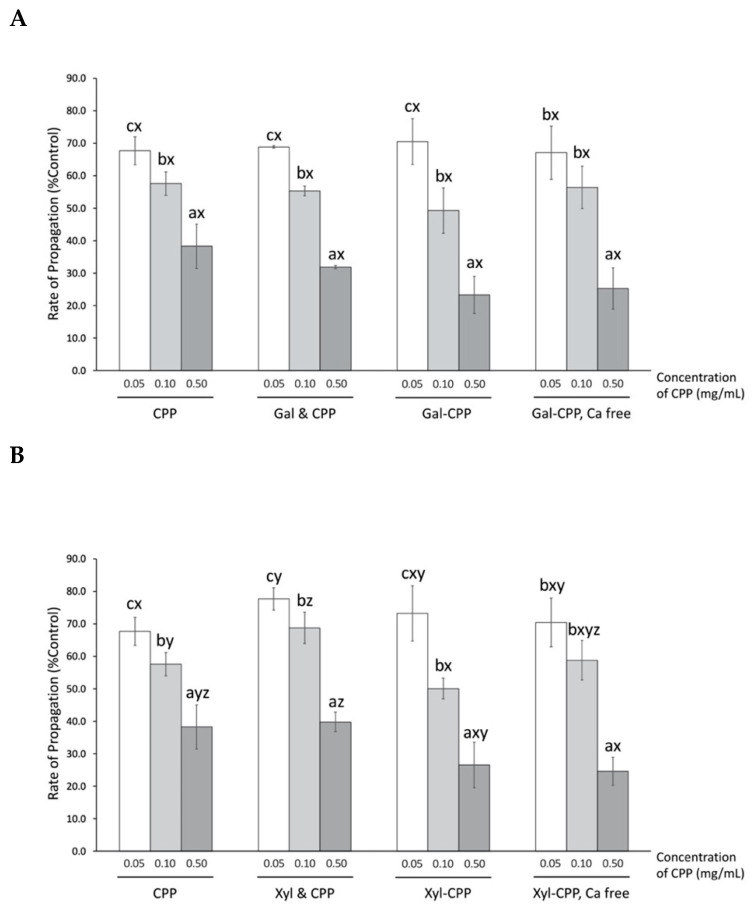
Rate of propagation of AAPH-induced soybean lecithin liposome peroxidation by CPP and CPP–polysaccharide ((**A**) Gal; and (**B**) Xyl) conjugates and mixtures as percentage of that by control (percent control). Data from three individual experiments were submitted to linear regression, and the rate of propagation was calculated as the slope of regression equation. CPP, casein phosphopeptide; Gal (or Xyl) & CPP, non-conjugated mixtures of galactomannan (or xyloglucan) and CPP; Gal–CPP or Xyl–CPP, conjugates of galactomannan or xyloglucan with CPP formed by employing Maillard reaction conditions. All results are expressed as mean ± SD (*n* = 3) and were statistically analyzed by one-way ANOVA and Tukey’s post-hoc test. Letters (a–c) denote significantly different means within the same treatment group, indicating effects of CPP concentration; letters (x–z) denote significantly different means within the same CPP concentration in an assay, indicating effects of treatment (*p* < 0.01).

The presence of CPP significantly (*p* < 0.01) lowered the propagation of peroxyl radical-induced damage of liposomes in a concentration-dependent manner—at the highest concentration tested for this assay (0.50 mg/mL), CPP was the most potent at decreasing peroxyl radical-induced damage on liposomes to 38.3% of control (Figure 5), which is comparable to the 1.0 μg/mL Trolox tested as a positive control (32.0% control). The presence of Gal or Xyl alone had no effect on reducing AAPH-induced peroxyl radical (data not shown). Thus, non-conjugated mixtures of CPP with Gal or Xyl present, respectively, also did not affect antioxidant activity. Moreover, conjugation of CPP to either Gal or Xyl was also limited to further enhance the ability of CPP to suppress liposome oxidation. Calcium-free CPP conjugates did not improve the protection offered by CPP against liposome lipid oxidation.

## 4. Discussion

Pure amino acid preparations derived from protein hydrolysates have a low ability to protect against lipid oxidation, thus implicating the importance of peptide linkages in contributing to observed antioxidant activity [6]. Casein has been shown to exhibit notable antioxidant activity in both the native state [50,51], as well as crude [8,19] and purified low molecular weight phosphopeptide fractions [9,10,21]. There are numerous examples of different food protein-derived byproducts from enzymatic hydrolysis during food processing that exhibit improved antioxidant activities, including animal and plant protein hydrolysates [52,53,54,55]. These derived products provide protection against oxidation reactions that result in improved product shelf-life when incorporated into the formulation or added to the packaging material. Former studies have shown milk casein to display a relatively higher surface activity and superior adhesion to oil-in-water emulsions compared to other protein sources that include whey protein β-lactoglobulin, egg yolk phosvitin, and soy protein β-conglycinin [37,38], which in turn could contribute to greater antioxidant properties [56]. The amphiphilic nature and surface-active behavior attributed to the flexibility of the peptide backbone contribute to the hydrophobic–hydrophilic balance.

The effect of this physiochemical property on antioxidant activity was tested with CPP and CPP-conjugates with polysaccharides, such as galactomannan (Gal) or xyloglucan (Xyl) in this experiment, to determine if CPP antioxidant activity could be improved in both aqueous (water–water interface) and emulsion (oil–water interface) model systems. Galactomannan is a polysaccharide composed of a β-1,4-D-mannose backbone with varying numbers of α-1,6 linked branches of a single α-D-galactose unit [57]. Xyloglucan is another polysaccharide, derived from tamarind seed, that has a β-1,4-D-glucan backbone substituted with α-1,6 linked side chains of α-D-xylose and α-1,2 linked β-D-galactosyl units to glucose residues [58]. We hypothesized that the hydrophilic character of these polysaccharides if successfully conjugated to CPP could result in an increase surface activity and possibly conformational changes of peptide structure, that would alter interfacial adsorptive and hydrophilicity, thus enhancing the antioxidant activity of CPP.

Preparation of CPP–polysaccharide conjugates involved first tryptic hydrolysis of bovine casein at the carboxyl ends of L-arginine or L-lysine residues on the protein [59]. The free lysine ε-amino group of CPP served as the attachment site for the reducing ends of polysaccharide, which was conjugated by a controlled Maillard reaction [60].

A specific motif consisting of three phosphoserine followed by two glutamate residues, Ser(P)-Ser(P)-Ser(P)-Glu-Glu, are commonly present in all CPPs, and thought to be essential for cationic metal binding [61]. With a relative high content of phosphoseryl residues in the proximity of hydrophobic residues such as valine, isoleucine, leucine, and proline, CPPs possesses a distinct amphipathic nature with both polar and hydrophobic domains. CPPs also possess a highly flexible backbone that allows residues remote from the multi-phosphorylated domain to also interact with metal ions [62].

The potential antioxidant activity of the CPP fraction used in this study, and associated conjugates and mixtures with polysaccharides, was assessed in both polar and non-polar medium. In the case of the ascorbate-mediated Fenton reaction in aqueous conditions, generation of hydroxyl radical will result in degradation of deoxyribose. In the non-site-specific binding assay, the presence of chelator EDTA (Fe^2+^-EDTA + H_2_O_2_) favors oxidation of ferrous iron and the generation of hydroxyl radicals (^•^OH). In the site-specific binding assay, EDTA is absent, allowing ferrous iron (Fe^2+^ + H_2_O_2_) to interact directly with deoxyribose before proceeding to generate hydroxyl radicals from Fenton reaction. These two assays were used to determine the affinity of CPP to scavenge ^•^OH radicals and to sequester Fe^2+^, respectively.

We confirmed from our earlier study that CPP derived from tryptic digest of casein has the capacity to not only inhibit oxidative damage of deoxyribose through sequestering the catalytic, pro-oxidant ferrous ions as predicted, but also impede the initiation of deoxyribose oxidation by stabilizing ^•^OH radicals which otherwise would attack deoxyribose [9]. With this mechanism clearly defined, we proposed that the hydrophilic character of polysaccharides would improve the solubility of CPP upon conjugation and thus enhance CPP antioxidant activity in an aqueous model. However, conjugation did not improve the affinity for CPP to exert an enhanced antioxidant activity by quenching hydroxyl radicals or binding to reactive ferrous ion. In contrast, the polysaccharides marginally reduced the natural antioxidant activity of CPP. The conjugation of CPP with Gal retained CPP antioxidant activity to a relatively greater extent than the CPP conjugate with Xyl, a shorter-chain polysaccharide. This suggested that saccharide chain length, a factor influencing the Maillard conjugation reaction, was a factor in predicting the antioxidant capacity of the conjugate product. In addition, Xyl was reported to be highly prone to ^•^OH attack [63], thus posing a potential vulnerability of Xyl–CPP conjugate to free radical damage which in turn would weaken the antioxidant activity of the glycoprotein conjugate. Alternatively, the addition of polysaccharides to the CPP backbone may have also altered the peptide configuration by disrupting the β loops and turns [62] of the multi-phosphorylated sections of CPP, thus exposing and rendering the reactive domain less effective. Thus, the distortion in CPP conformation by glycosylation may have reduced direct interaction with Fenton pro-oxidant constituents, thus impairing antioxidant activity derived from CPP–polysaccharide conjugates, compared to native CPP. The removal of calcium was found to reduce CPP activity to inhibit deoxyribose degradation, likely by affecting solubility of CPP, which otherwise would be higher in the presence of calcium by forming soluble complexes [61].

In addition to the deoxyribose Fenton reaction model, an additional aqueous model was used to confirm the radical scavenging activity of CPP, CPP–polysaccharide conjugates and mixtures. Primary antioxidant activity of CPP and associated conjugates was measured by the extent of reducing radical monocation (ABTS^•+^) by hydrogen/electron donating activity [64]. Conjugation of CPP with Gal and Xyl did not affect CPP antioxidant activity, but removing calcium from the Gal–CPP conjugate significantly enhanced antioxidant functionality. The ABTS^•+^ radical is positively charged and electrostatically similar to calcium cation (Ca^2+^), hence a possible charge competition for the negatively charged functional CPP domain would explain the reduced functionality to scavenge free radicals.

Aside from aqueous models, the radical scavenging activity of CPP and conjugates were tested in an emulsion model using liposomes. Thermolysis of heat-labile azo compounds such as AAPH will generate a low but continuous flux of peroxyl radicals [65]. Soybean lecithin (phosphatidylcholine) liposomes have peroxidizable membranes [66] that propagate the peroxidation reactions of AAPH-derived radicals generated in the membrane phase [67]. The extent of antioxidant activity of CPP and associated conjugates is limited by the chain-breaking affinity that localizes at the liposomal membrane surface (i.e., oil–water interface), the site of radical-generated oxidation. Covalent and non-covalent interactions between surface-active peptides and saccharides will result in steric stabilization where the hydrophilic saccharide segments are solvated in the aqueous phase, while the hydrophobic peptide residues are anchored in the surface of oil droplets [68]. The change in surface activity should place the peptide–saccharide immediately adjacent to the emulsion interface [69], where free radical oxidations primarily occur [70].

The amphiphilic property of CPP provides an ideal conformation for localization at the oil–water interface where it could quench peroxyl radicals and thus explain the significant reduction in propagation rate reactions compared to control, in the AAPH assay (Figure 5). Conjugated or a simple mixture of CPP with highly hydrophilic polysaccharides did not improve the affinity of CPP to localize at the liposome membrane and express the antioxidant activity thereof. The disruption of ideal CPP configuration upon addition of polysaccharides is speculated to be the reason. Furthermore, removal of calcium from CPP conjugates had no effect on the rate of propagation, suggesting the independence of calcium content on surface activity of CPP to scavenge peroxyl radicals.

It has been previously shown that conjugation of galactomannan to whole proteins, such as casein [71], ovalbumin [33], and lysozyme [32,34,72], would enhance protein functionality and antioxidant capacity. However, in the case of CPP, a product of casein trypsin hydrolysis, being smaller likely explains why conjugation was not as successful, as expected when applied to whole globular proteins. Our results show that the polar multi-phosphoseryl domain, which is a characteristic requirement for metal chelating affinity and also free radical scavenging for CPP in both aqueous and emulsion conditions, does not require further modification to enhance antioxidant activity.

## 5. Conclusions

Results of this study confirm the fact that CPP has the capacity to reduce deoxyribose oxidation, likely by binding ferrous iron and sequestering hydroxyl radicals that are generated from Fenton reactants. The CPP complex was also effective at quenching peroxyl radicals in the liposome peroxidation model. Conjugation of CPP with galactomannan and xyloglucan did not enhance these confirmed mechanisms of CPP antioxidant activity. These observations showed that CPP serves effectively as both a primary and secondary antioxidant, without the need for further modifications. CPPs represent a potential bioactive ingredient that could be used to preserve overall food quality.
